# A Systematic Review of Micropulse Laser Trabeculoplasty (MLT) in Primary Open-Angle Glaucoma (POAG) Management: Efficacy, Safety, and Future Perspectives

**DOI:** 10.3390/biomedicines13010211

**Published:** 2025-01-16

**Authors:** Filippo Confalonieri, Barbara Casarini, Annalaura Papapicco, Fabio Stiro, Raffaele Piscopo, Luca D’Andrea, Goran Petrovski, Tommaso Verdina

**Affiliations:** 1 Department of Ophthalmology, IRCCS Humanitas Research Hospital, Rozzano, 20089 Milan, Italy; 2 Institute of Ophthalmology, University of Modena and Reggio Emilia, Azienda Ospedaliero-Universitaria di Modena, 41122 Modena, Italy; barbara.casarini@gmail.com (B.C.); annalaura.papapicco@unipr.it (A.P.); tommaso.verdina@gmail.com (T.V.); 3 Ophthalmology Unit, University Hospital of Parma, 43126 Parma, Italy; 4 Eye Unit, Vimercate Hospital, 20871 Vimercate, Italy; fabiostiro@gmail.com; 5 Eye Clinic, Department of Neurosciences, Reproductive and Odontostomatological Sciences, University of Naples Federico II, Via Pansini n. 5, 80131 Naples, Italy; piscoporaffaele@tin.it (R.P.); dandrea.luca91@gmail.com (L.D.); 6 Center for Eye Research and Innovative Diagnostics, Department of Ophthalmology, Institute for Clinical Medicine, University of Oslo, Kirkeveien 166, 0450 Oslo, Norway; goran.petrovski@medisin.uio.no; 7 Department of Ophthalmology, Oslo University Hospital, Kirkeveien 166, 0450 Oslo, Norway; 8 Department of Ophthalmology, University of Split School of Medicine and University Hospital Centre, 21000 Split, Croatia; 9 UKLONetwork, University St. Kliment Ohridski-Bitola, 7000 Bitola, North Macedonia

**Keywords:** glaucoma, micropulse laser trabeculoplasty (MLT), minimally invasive glaucoma surgery, minimally invasive bleb surgery, non-plate, bleb-forming glaucoma devices, trabeculectomy, glaucoma-therapy-related ocular surface disease, glaucoma drainage devices, non-penetrating glaucoma surgery, ciliary body function modulation, selective laser trabeculoplasty

## Abstract

Background/Objectives: Micropulse laser trabeculoplasty (MLT) is gaining attention as a non-invasive treatment option for primary open-angle glaucoma (POAG), offering an alternative to traditional surgeries and medications. This systematic review evaluates the effectiveness, safety, and potential of MLT in glaucoma management. Methods: This review adhered to the Preferred Reporting Items for Systematic Reviews and Meta-Analyses (PRISMA) guidelines. The strength of evidence was assessed using the Grading of Recommendations Assessment, Development, and Evaluation (GRADE) methodology, following the 2011 Oxford Centre for Evidence-Based Medicine (OCEBM) recommendations. Results: We identified 79 articles, and after removing duplicates and screening abstracts, 56 articles were eligible for further review. A detailed full-text analysis was conducted on 26 articles, of which 15 met the predefined inclusion criteria. Conclusions: MLT shows promise as a primary or adjunctive treatment for reducing intraocular pressure (IOP) in glaucoma and ocular hypertension patients. Current evidence supports its efficacy and safety; however, additional long-term studies are needed to confirm its durability and compare its effectiveness with traditional surgical and pharmacological approaches. Standardizing treatment protocols and refining patient selection criteria could enhance MLT’s clinical value and support its broader adoption in glaucoma care.

## 1. Introduction

Glaucoma is a leading cause of irreversible blindness worldwide, characterized by progressive optic nerve damage and visual field loss [1]. Elevated intraocular pressure (IOP) is a major modifiable risk factor in glaucoma pathogenesis. While pharmacotherapy remains the cornerstone of glaucoma management, surgical interventions such as trabeculectomy and minimally invasive glaucoma surgeries (MIGSs) are often pursued in cases of inadequate IOP control or intolerance to medications.

Micropulse laser trabeculoplasty (MLT) has emerged as a non-invasive, repeatable treatment option for reducing IOP by enhancing aqueous outflow through the trabecular meshwork. Unlike traditional continuous-wave lasers, MLT delivers energy in short, repetitive pulses interspersed with cooling intervals. This approach minimizes thermal damage to the trabecular meshwork while promoting aqueous humor outflow.

The mechanisms of MLT involve several principles. First, MLT stimulates trabecular meshwork endothelial cells, enhancing aqueous humor outflow [2]. Second, the cooling intervals between pulses reduce collateral thermal damage to adjacent tissues [3]. Third, MLT induces biological modulation, including cytokine release and extracellular matrix remodeling, which contributes to sustained IOP reduction [4].

Selective laser trabeculoplasty (SLT) is another established laser treatment for IOP control. It employs a Q-switched frequency-doubled 532 nm Nd:YAG laser that targets pigmented trabecular cells with short bursts of energy [5]. In contrast, MLT uses a 532 nm green, a 577 nm yellow, or an 810 nm diode laser in a micropulse delivery mode. The cooling intervals in MLT minimize thermal damage, reducing inflammation and the risk of complications. Conversely, SLT produces microthermal effects at the cellular level, which can result in pigment dispersion and mild inflammation within trabecular meshwork cells, occasionally leading to intraocular pressure spikes [6,7,8].

This systematic review aims to consolidate the current evidence regarding the efficacy, safety, and future directions of MLT in glaucoma management.

## 2. Materials and Methods

A systematic search of the literature was conducted across PubMed, Embase, Scopus, and Cochrane Library databases to identify studies published up to 15 February 2024. The search strategy incorporated keywords such as “micropulse laser trabeculoplasty”, “MLT”, “glaucoma”, “intraocular pressure”, and “ocular hypertension”. Eligible study types included randomized controlled trials (RCTs), prospective cohort studies, retrospective analyses, and systematic reviews.

Studies were selected based on predefined eligibility criteria, focusing on interventions involving MLT for glaucoma treatment, outcomes related to intraocular pressure (IOP) reduction, safety profiles, and follow-up duration. Inclusion criteria required studies to link the micropulse laser trabeculoplasty (MLT) technique with IOP reduction in primary open-angle glaucoma (POAG). Studies reporting outcomes unrelated to IOP reduction with MLT, as well as review articles, pilot studies, case series, case reports, photo essays, and studies written in languages other than English, were excluded. Additionally, studies involving animal eyes, cadaveric eyes, and pediatric patients were excluded.

Data extraction and synthesis were independently performed by two reviewers (T.V. and F.S.), with discrepancies resolved through the consensus of a third senior consultant (F.C.). The search was limited to studies published in English but was not restricted by publication type, study design, or publication date. The complete search strategy is provided in Appendix A.

The strength of the evidence was evaluated using the Grading of Recommendations Assessment, Development, and Evaluation (GRADE) approach, following the 2011 Oxford Centre for Evidence-Based Medicine (OCEBM) recommendations (Table 1).

## 3. Results

**Figure 1:**
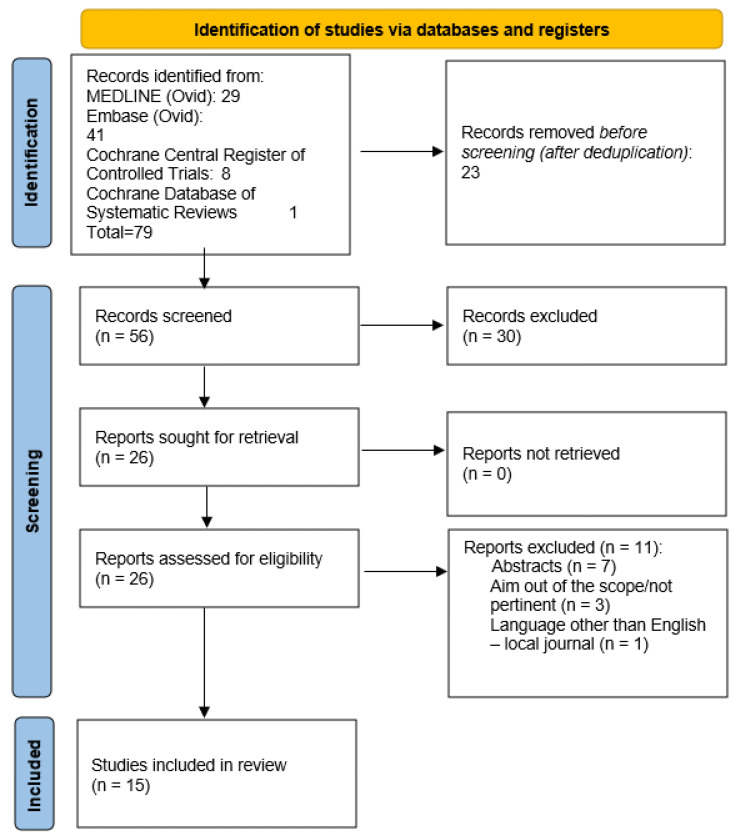
Flowchart of the literature search and selection according to the Preferred Reporting Items for Systematic Reviews and Meta-Analyses guidelines (PRISMA).

A total of 79 articles were identified. Following the removal of duplicates and screening of abstracts, 56 articles remained. Subsequently, a comprehensive full-text review of 26 articles was conducted. Ultimately, 15 articles met the predefined inclusion criteria (Appendix B). All the studies evaluated the efficacy of MLT in primary open-angle glaucoma (POAG), pseudoexfoliative glaucoma (PXG), and ocular hypertension (OHT). Across various study designs, MLT consistently demonstrated a significant reduction in IOP. Adverse events associated with MLT were generally mild and transient, including intraocular inflammation, transient IOP spikes, and corneal epithelial defects. Long-term follow-up data on the durability of IOP reduction post-MLT are limited but suggest sustained efficacy over months to years. Figure 1 summarizes the research approach applied in this systematic review within a flowchart.

The specific papers with determining reasons for inclusion or exclusion of the full-text reviewed articles are reported in Appendix B.

## 4. Discussion

Laser trabeculoplasty techniques have long been recognized as safe, non-invasive, and repeatable methods for reducing intraocular pressure (IOP) in glaucoma patients. Micropulse laser trabeculoplasty (MLT), introduced in 2005, employs a 15% duty cycle targeting the anterior trabecular meshwork. This technique minimizes thermal tissue damage, postoperative inflammation, and peripheral anterior synechiae (PAS) formation compared to conventional laser trabeculoplasty (CLT) [21].

Several studies have assessed the efficacy and safety of MLT. In 2015, Lee et al. conducted a prospective cohort study on 48 eyes with primary open-angle glaucoma (POAG), including normal tension glaucoma (IOP < 21 mm Hg). A single session of 360° MLT significantly reduced IOP at all follow-up intervals up to six months and decreased glaucoma medication use. At the six-month follow-up, 20% of patients achieved IOP lowering and 21% reduced their medication use. No significant side effects were observed [16].

In a retrospective review by Valera-Cornejo et al., in 2018, 30 eyes treated with 360° MLT showed a ≥ 20% IOP reduction in 35% and 41% of cases at three and six months, respectively. A trend toward greater IOP reduction with higher baseline IOP was observed, although this was not statistically significant due to the small sample size [13].

In 2019, Hong et al. evaluated the outcomes of MLT in 72 eyes, reporting a 20% IOP decrease from baseline across a 24-week follow-up. The study also found reduced medication use and no significant inflammation [10]. Another study by Phan et al. in 2019 stratified outcomes based on baseline IOP and glaucoma severity. Patients with higher initial IOP and early-stage glaucoma experienced significant IOP reductions, while those with advanced glaucoma did not [18].

Yang et al., in 2022, documented sustained IOP reductions at one day, three months, and six months post-treatment in 39 eyes. However, at 36 months, only 21.88% of patients achieved a ≥ 20% reduction, indicating MLT’s diminishing efficacy over time [20].

The role of operator expertise has also been highlighted. In a study by Kakihara et al., MLT efficacy was greater when performed by experienced glaucoma specialists. This underscores the need for further investigation into the impact of operator expertise on treatment outcomes [11].

MLT has also been evaluated in pseudoexfoliation glaucoma (PEXG). In 2019, Makri et al. treated 27 PEXG eyes with inadequate IOP control using prostaglandin analogs, reporting significant IOP reductions up to 12 months, with 52.17% of cases achieving a ≥20% reduction [17]. Similarly, Aydin Kurna et al. assessed 180° MLT in 51 eyes with uncontrolled POAG or PEXG, achieving IOP reduction in 34–42% of cases during follow-ups of up to 48 months. A significant correlation was observed between baseline IOP and treatment success, while age and laser power showed no correlation [14].

Comparisons between MLT and selective laser trabeculoplasty (SLT) reveal distinct advantages and limitations. Hirabayashi et al. reported similar success rates for MLT (44%) and SLT (40%) in a cohort of 100 eyes. MLT showed consistent efficacy regardless of age or baseline IOP and did not induce IOP spikes, unlike SLT [9,22]. Pimentel et al. found SLT to be slightly more effective at 12 months in achieving an IOP ≤ 21 mm Hg with ≥20% reduction, although MLT demonstrated comparable overall efficacy [6]. Sun et al. observed greater IOP reductions with SLT at early follow-ups, but similar long-term outcomes between the two techniques [19]. Table 2 summarizes the differences between MLT and SLT in POAG treatment.

Studies suggest that MLT may cause less inflammation and fewer IOP spikes post-treatment compared to SLT, making it a safer option for some patients [5,8].

In conclusion, MLT presents a promising, less invasive alternative to traditional methods for managing glaucoma and ocular hypertension, particularly in patients at risk of post-treatment complications. However, further research is needed to optimize treatment protocols, assess long-term efficacy, and clarify inter-operator variability.

Although there is now sufficient evidence to consider MLT a safe and effective treatment for glaucoma patients, several areas remain worthy of further investigation. These include determining optimal treatment parameters such as laser energy settings, the number of laser applications, and retreatment intervals. Additionally, many studies conducted to date have limitations that may affect their results. These include retrospective designs that may introduce selection bias, small sample sizes, short follow-up periods, a lack of randomization, and the absence of control groups in some cases.

Patient selection criteria, including baseline IOP, glaucoma subtype, and prior treatment history, may also influence the likelihood of treatment success. Therefore, future studies should aim to include more homogeneous cohorts to enhance the reliability of findings.

Future research directions should focus on prospective, comparative studies to elucidate the mechanisms underlying MLT’s IOP-lowering effects, explore combination therapy approaches, and assess the cost-effectiveness of MLT compared to standard treatments.

## 5. Conclusions

MLT represents a valuable adjunctive or primary treatment option for lowering IOP in patients with glaucoma or ocular hypertension. While existing evidence supports its efficacy and safety profile, further long-term studies are warranted to establish its durability and comparative effectiveness relative to traditional surgical and pharmacological interventions. The standardization of treatment protocols and refinement of patient selection criteria may enhance the clinical utility of MLT and pave the way for its widespread adoption in glaucoma management.

One key area of future research is the identification of patient profiles most likely to benefit from MLT. While current evidence supports its use across various glaucoma subtypes, differences in individual responses suggest that factors such as baseline intraocular pressure, angle anatomy, and disease progression rates may influence outcomes. Longitudinal studies with diverse patient populations are needed to develop predictive models for tailoring MLT to individual needs.

Another promising avenue for investigation is the integration of MLT with pharmacological and surgical interventions. Understanding the synergistic effects of MLT with medications such as prostaglandin analogues or its role as a bridge therapy before more invasive procedures could optimize treatment algorithms. Randomized controlled trials should evaluate the efficacy and safety of such combination approaches, particularly in patients with advanced or refractory glaucoma.

Finally, economic considerations are critical for the widespread adoption of MLT. Comprehensive cost-effectiveness analyses comparing MLT with other treatment modalities, including traditional laser trabeculoplasty and newer surgical options, are essential. These studies should consider not only direct costs but also the long-term economic impact of reduced medication use and delayed progression to invasive surgeries.

## Figures and Tables

**Table 1:** Characteristics, quality, and level of evidence of the included studies.

Author	Year	Study Design	Study Sample (Eyes)	Entity of IOP Reduction in MLT	Comparison to Other Treatments	Grade	Level
Hirabayashi et al. [9]	2019	Retrospective review	50 MLT-treated eyes and 50 SLT-treated eyes	MLT: 2.1 ± 4.1 mm Hg; SLT: 1.8 ± 6.6 mm Hg at 6 mo	MLT had similar efficacy to SLT but fewer IOP spikes	Moderate	III
Hong et al. [10]	2019	Retrospective study	72 eyes of 72 POAG patients	Pre-MLT: 20.6 ± 5.9 mm Hg; post-MLT (6 mo): 16.5 ± 2.9 mm Hg	No IOP spikes after MLT, suggesting MLT is safe and effective for POAG	Moderate	III
Kakihara et al. [11]	2021	Retrospective study	42 eyes of 34 OAG patients	Pre-MLT: 19.1 ± 6.5 mm Hg; post-MLT (6 mo): 13.1 ± 5.0 mm Hg	Higher success rate if performed by experienced specialists; no IOP spikes after MLT	Moderate	III
Pimentel RL et al. [6]	2023	Comparative retrospective study	SLT group (45 eyes) and MLT group (37 eyes)	Mean IOP reductions from baseline in MLT groups (−5.8 ± 2.6 mm Hg, 23.4%)in the SLT (−6.0 ± 3.3 mm Hg, 24.9%) (*p* = 0.74)	MLT success rate: 58.7%; SLT success rate: 61.5%	Moderate	III
Rantala et al. [12]	2012	Retrospective study	40 eyes of 29 OAG patients	Pre-MLT: 21.8 ± 4.9 mm Hg; post-MLT (6 mo): 19.2 mm Hg	MLT is safe but ineffective; 97.5% failure rate for IOP (reduction by 20%)	Low	III
Robin et al. [8]	2023	Comparative retrospective study	54 eyes of 99 OAG or OHT patients	Pre-MLT: 22.8 ± 4.00 mm Hg; post-MLT (1 year): <3 mm Hg or <20% in 48% of cases	MLT has a lower incidence of IOP spikes compared to SLT and a similar success rate at 1 year	High	II
Valera-Cornejo et al. [13]	2018	Retrospective study	30 eyes of OAG patients	Pre-MLT: 15.6 ± 3.5 mm Hg; post-MLT (last follow-up): 12.8 ± 2.6 mm Hg	No IOP spikes; MLT slightly reduces IOP for a short time	Moderate	III
Abramowitz et al. [5]	2018	Prospective randomized study	69 eyes of OAG patients	Pre-MLT: 18.26 mm Hg; post-MLT (1 h): 15.15 mm Hg	MLT and SLT have similar efficacy; MLT has less discomfort during and after the procedure	High	I
Aydin Kurna S et al. [14]	2022	Retrospective study	51 eyes of POAG and PXE glaucoma patients	Pre-MLT: 22.69 ± 3.1 mm Hg; post-MLT (last follow-up): 19.08 ± 2.98 mm Hg at 12 months	No significant difference in mean IOP reductions and success rates at 12 months between MLT and SLT	Low	III
Babalola et al. [15]	2015	Retrospective study	30 eyes of 16 Nigerian patients with POAG	Pre-MLT: 18.6 mm Hg; post-MLT (1 h): 15.5 mm Hg; immediate reduction: 3.2 mm Hg (17.2%)	No serious side effects, confirming similar results in Western populations	Low	III
Lee et al. [16]	2015	Prospective cohort study	48 eyes of OAG patients	Pre-MLT: 18.5 ± 3.0 mm Hg; post-MLT (6 mo): 14.9 ± 2.5 mm Hg (19.5% reduction)	MLT reduces IOP and medication use, similar to SLT but with less inflammation	High	II
Makri et al. [17]	2018	Prospective single-center one-arm study	27 eyes of PEXG patients	Pre-MLT: 20.41 ± 1.87 mm Hg; post-MLT (12 mo): 15.74 ± 0.96 mm Hg (21.51% reduction)	MLT effectively reduces IOP in PEXG patients for 12 months without significant complications	Moderate	II
Phan et al. [18]	2021	Retrospective study	34 eyes of 19 POAG and OHTN patients	Pre-MLT: 17.47 ± 3.59 mm Hg; post-MLT (3 mo): 16.15 ± 3.75 mm Hg (7.6% reduction)	MLT is effective in reducing IOP, especially in patients with IOP >16 mm Hg and early glaucoma	Moderate	III
Sun et al. [19]	2021	Retrospective comparative cohort study	43 MLT-treated eyes and 85 SLT-treated eyes	Pre-MLT: 18.0 mm Hg; post-MLT (1y): 16.7 mm Hg (7.2% reduction)	MLT has fewer IOP spikes compared to SLT and similar overall efficacy	High	II
Yang et al. [20]	2022	Prospective single-center study	39 eyes of glaucoma or OHT patients	Pre-MLT: 21.13 ± 7.75 mm Hg; post-MLT (6 mo): 17.52 ± 4.25 mm Hg (12.0% reduction)	MLT reduces IOP significantly up to 6 months, with limited long-term efficacy	Moderate	II

**Table 2:** Advantages of MLT in comparison to SLT.

Characteristic	MLT	SLT
Mechanism	Micropulse energy with cooling intervals	Microthermal effects on pigmented cells
Efficacy	Comparable to SLT	Proven effectiveness
Safety	Lower inflammation; fewer complications	Mild inflammation possible
Repeatability	High; no cumulative damage	High but limited by potential effects
Recovery	Quicker recovery; less discomfort	May require anti-inflammatory treatment

## Data Availability

All the data are available on reasonable request from the corresponding author.

## Appendix A

**Documentation on the literature search**

Micropulse laser trabeculoplasty (MLT) was used for primary open-angle glaucoma (POAG).

The following databases were searched:

**Database**	**Number of Retrieved References**
MEDLINE (Ovid)	29
Embase (Ovid)	41
Cochrane Central Register of Controlled Trials	8
Cochrane Database of Systematic Reviews	1
Number of references before deduplication	79
Number of references after deduplication	56

All searches were conducted on 15 February 2024 by Sølvi Biedilæ, a senior librarian, at the Library of Medicine and Science, University of Oslo.

**Ovid Databases**	
exp/	Exploded index term
/	After an index, the term indicates that a subject heading is selected
.tw,kf.	This searches for a term in the title, abstract, and author keywords
*	This truncates the end of a term; for example, diet * retrieves both diet, diets, and dietary
Adj3	This searches for two terms next to each other, in any order, with up to three words in between
**Cochrane Library**	
NEAR/3	This searches for two terms next to each other, in any order, with up to three words in between

**Ovid MEDLINE(R) ALL <1946 to 14 February 2024>**

1.	exp Glaucoma/	60,993
2.	glaucoma*.tw,kf.	73,555
3.	1 or 2	84,174
4.	((micropulse* or micro pulse*) adj3 laser* adj3 trabecul*).tw,kf.	29
5.	mlt.tw,kf.	1444
6.	4 or 5	1457
7.	3 and 6	29

**Embase Classic+Embase <1947 to 2024 February 14>**

1.	exp Glaucoma/	116,701
2.	glaucoma*.tw,kf.	99,413
3.	1 or 2	130,841
4.	((micropulse* or micro pulse*) adj3 laser* adj3 trabecul*).tw,kf.	38
5.	mlt.tw,kf.	1889
6.	4 or 5	1904
7.	3 and 6	41

**Cochrane Library**

ID	Search	Hits
#1	MeSH descriptor: [Glaucoma] explode all trees	4180
#2	(glaucoma*)	9377
#3	#1 or #2	9377
#4	((micropulse* or micro pulse*) near/3 laser* near/3 trabecul*)	9
#5	(mlt)	169
#6	#4 or #5	173
#7	#3 and #6 in Cochrane Reviews	1
#8	#3 and #6 in Trials	8

## Appendix B

**Table A1:** Second round of papers inclusion with reasons behind the choices.

**Paper (Total 26; 15 Included; 11 Excluded)**	**Included (Comment)**	**Excluded (Reason)**
Hirabayashi, M. T., Rosenlof, T. L., & An, J. A. (2019). Comparison of successful outcome predictors for MicroPulse^®^ laser trabeculoplasty and selective laser trabeculoplasty at 6 months. Clinical Ophthalmology, 13, 1001–1009. https://doi.org/10.2147/OPTH.S205977	A comparison of efficacy between SLT and MLT is provided.	
2. Hong Y, Song SJ, Liu B, Hassanpour K, Zhang C, Loewen N. Efficacy and safety of micropulse laser trabeculoplasty for primary open angle glaucoma. Int J Ophthalmol 2019;12(5):784-788	MLT is effective and safe for POAG patients. No patient experienced IOP spikes after MLT. The IOP 6 mo after treatment decreased significantly with less glaucoma medication.	
3. Kakihara S, Hirano T, Imai A, Kurenuma T, Chiku Y, Murata T. Micropulse laser trabeculoplasty under maximal tolerable glaucoma eyedrops: treatment effectiveness and impact of surgical expertise. Int J Ophthalmol. 2021 Mar 18;14(3):388-392. doi: 10.18240/ijo.2021.03.09. PMID: 33747814; PMCID: PMC7930551.	The 6-month effectiveness of MLT for controlling IOP is relatively limited in eyes with OAG using maximal tolerable glaucoma eyedrops. However, its effectiveness may be improved if performed by a glaucoma specialist with sufficient MLT experience.	
4. Pimentel RL, Alves Júnior RR, Lima WMML, Dantas LOR, Costa VP. Selective laser trabeculoplasty versus micropulse laser trabeculoplasty for intraocular pressure control in patients with primary open angle glaucoma: a 12-month retrospective comparative study. Lasers Med Sci. 2023 Apr 17;38(1):102. doi: 10.1007/s10103-023-03771-9. PMID: 37067669.	Both the original article (retrospective comparative study) and review are included.	
5. Rantala E, Välimäki J. Micropulse diode laser trabeculoplasty—180-degree treatment. Acta Ophthalmol. 2012 Aug;90(5):441-4. doi: 10.1111/j.1755-3768.2010.02026.x. Epub 2010 Nov 4. PMID: 21054817.	The results suggest that 180° MDLT is a safe but ineffective treatment in patients with open-angle glaucoma.	
6. Robin AZ, Syar P, Darwish D, Thomas C, Pfahler NM, Kakouri A, Patrianakos T, Giovingo M. Comparison of success rate and intraocular pressure spikes between selective laser trabeculoplasty and micropulse laser trabeculoplasty in African American and Hispanic patients. Int J Ophthalmol. 2023 Jan 18;16(1):75-80. doi: 10.18240/ijo.2023.01.11. PMID: 36659950; PMCID: PMC9815972.	MLT has a significantly lower incidence of pressure spikes and a similar treatment failure rate at 1-year post-procedure, demonstrating that it is a reasonable alternative compared to SLT.	
7. Valera-Cornejo DA, Loayza-Gamboa W, Herrera-Quiroz J, Alvarado-Vlllacorta R, Cordova-Crisanto L, Valderrama-Albino V, Davalos NP. Micropulse Trabeculoplasty in Open Angle Glaucoma. Adv Biomed Res. 2018 Dec 19;7:156. doi: 10.4103/abr.abr_203_17. PMID: 30662885; PMCID: PMC6319042.	MLT slightly reduces the IOP in a few patients with uncontrolled OAG for a very short time and may not be suitable for these patients.	
Enyr Saran Arcieri, Rafael Saran Arcieri; MICROPULSE DIODE LASER TRABECULOPLASTY RESULTS IN TREATMENT OF PRIMARY OPEN-ANGLE GLAUCOMA PATIENTS. Invest. Ophthalmol. Vis. Sci. 2014;55(13):6165.		ARVO abstract
antonella clemente, Caterina Toma, Stela Vujosevic, chiara padovan, stefano de cillà; Efficacy of Micropulse Laser Trabeculoplasty in Open Angle Glaucoma. Invest. Ophthalmol. Vis. Sci. 2019;60(9):696.		ARVO abstract
Peter Coombs, Nathan M Radcliffe; Outcomes of Micropulse Laser Trabeculoplasty vs. Selective Laser Trabeculoplasty. Invest. Ophthalmol. Vis. Sci. 2014;55(13):6155.		ARVO abstract
Robert G Dionisio, Olga Lekakh German, Thomas Patrianakos, Michael Giovingo; MLT vs. SLT in the Hispanic and African American Population for Treatment of Open-Angle Glaucoma. Invest. Ophthalmol. Vis. Sci. 2018;59(9):6097.		ARVO abstract
Briana C. Gapsis, Matthew Bickford, Robert Allan Sharpe, Sudeep Das, Leah Kammerdeiner, Matthew J. Nutaitis; Analysis of the Relative Efficacy of Micropulse Laser Trabeculoplasty and Selective Laser Trabeculoplasty. Invest. Ophthalmol. Vis. Sci. 2018;59(9):6090.		ARVO abstract; outcome is out of the scope of the review
Kunjal K Modi, Scott M Walsman; Effect of Increasing Shot Number in Micropulse Laser Trabeculoplasty (MLT) for Open Angle Glaucoma. Invest. Ophthalmol. Vis. Sci. 2017;58(8):4976.		ARVO abstract; outcome is out of the scope of the review
Marcelle Morcos, Jack Klenda, Patricia Fortin, Andrew Pansick, Christian Draper; Impact of Micropulse Laser Trabeculoplasty: A 2-Year Retrospective Analysis. Invest. Ophthalmol. Vis. Sci. 2022;63(7):170–A0363.		ARVO abstract
D.D. Ingvoldstad, R. Krishna, L. Willoughby; MicroPulse Diode Laser Trabeculoplasty versus Argon Laser Trabeculoplasty in the Treatment of Open Angle Glaucoma. Invest. Ophthalmol. Vis. Sci. 2005;46(13):123.		ARVO abstract
8. Abramowitz B, Chadha N, Kouchouk A, Alhabshan R, Belyea DA, Lamba T. Selective laser trabeculoplasty vs. micropulse laser trabeculoplasty in open-angle glaucoma. Clin Ophthalmol. 2018 Aug 30;12:1599-1604. doi: 10.2147/OPTH.S167102. PMID: 30214144; PMCID: PMC6124459.	Micropulse trabeculoplasty has demonstrated similar efficacy to SLT over a 52-week follow-up period with less discomfort experienced both during and after the procedure.	
9. Aydin Kurna S, Sonmez AD, Yamic M, Altun A. Long-term results of micropulse laser trabeculoplasty with 577-nm yellow wavelength in patients with uncontrolled primary open-angle glaucoma and pseudoexfoliation glaucoma. Lasers Med Sci. 2022 Aug;37(6):2745-2752. doi: 10.1007/s10103-022-03550-y. Epub 2022 Mar 30. PMID: 35353248.	The reduction in intraocular pressure shows a significant correlation with baseline intraocular pressure, while age and laser power show no correlation (*p* > 0.05). MLT is a novel treatment option for patients with glaucoma with favorable long-term outcomes and a good safety profile.	
10. Babalola OE. Micropulse diode laser trabeculoplasty in Nigerian patients. Clin Ophthalmol. 2015 Jul 20;9:1347-51. doi: 10.2147/OPTH.S82678. PMID: 26229426; PMCID: PMC4516186.	Micropulse diode laser trabeculoplasty is a useful adjunct in the management of open-angle glaucoma in Nigerians. This corroborates the findings of other researchers in western populations.	
Detry-Morel M, Muschart F, Pourjavan S. Micropulse diode laser (810 nm) versus argon laser trabeculoplasty in the treatment of open-angle glaucoma: comparative short-term safety and efficacy profile. Bull Soc Belge Ophtalmol. 2008;(308):21-8. PMID: 18700451.		Language other than English and a local journal
11. Lee JWY, Yau GSK, Yick DWF, Yuen CYF. MicroPulse Laser Trabeculoplasty for the Treatment of Open-Angle Glaucoma. Medicine (Baltimore). 2015 Dec;94(49):e2075. doi: 10.1097/MD.0000000000002075. PMID: 26656331; PMCID: PMC5008476.	MLT is effective in reducing IOP and medications in OAG with minimal post-laser inflammation and low failure rates at 6 months following laser trabeculoplasty.	
Makri OE, Pagoulatos D, Kagkelaris K, Plotas P, Georgakopoulos CD. Evaluation of intraocular pressure in the first 24hours after micropulse laser trabeculoplasty in eyes with pseudoexfoliation glaucoma. J Fr Ophtalmol. 2019 Nov;42(9):983-986. doi: 10.1016/j.jfo.2019.05.008. Epub 2019 Jun 6. PMID: 31178072.		Studies with outcomes other than IOP lower the effect of MLT
12. Makri OE, Pallikari A, Pagoulatos D, Kagkelaris K, Kostopoulou EV, Georgakopoulos CD. Micropulse laser trabeculoplasty on pseuodexfoliation glaucoma patients under topical prostaglandin analogue monotherapy: 1-year results. Graefes Arch Clin Exp Ophthalmol. 2019 Feb;257(2):349-355. doi: 10.1007/s00417-018-4195-2. Epub 2018 Nov 28. PMID: 30488265.	Micropulse laser trabeculoplasty appears to be an effective method in lowering IOP in patients with PEXG up to 12 month of the follow-up period.	
13. Phan R, Bubel K, Fogel J, Brown A, Perry H, Morcos M. Micropulse laser trabeculoplasty and reduction of intraocular pressure: A preliminary study. Saudi J Ophthalmol. 2022 Feb 18;35(2):122-125. doi: 10.4103/1319-4534.337860. PMID: 35391804; PMCID: PMC8982934.	Patients with a higher initial IOP and in the early stages of glaucoma are more likely to benefit from MLT in lowering IOP. A randomized clinical trial is necessary to confirm these preliminary findings. We recommend that clinicians should consider MLT in the management of early glaucoma and among those with IOP >16 mm Hg.	
14. Sun CQ, Chen TA, Deiner MS, Ou Y. Clinical Outcomes of Micropulse Laser Trabeculoplasty Compared to Selective Laser Trabeculoplasty at One Year in Open-Angle Glaucoma. Clin Ophthalmol. 2021 Jan 22;15:243-251. doi: 10.2147/OPTH.S285136. PMID: 33519186; PMCID: PMC7837566.	Eyes have similar success after MLT compared to SLT at 1 year. Laser trabeculoplasty with either method can be offered as a treatment with consideration of MLT in those eyes where IOP spikes should be avoided.	
Catherine Q Sun, Yvonne Ou; Comparison of Outcomes of Micropulse Laser Trabeculoplasty versus Selective Laser Trabeculoplasty. Invest. Ophthalmol. Vis. Sci. 2018;59(9):6089.		ARVO abstract
15. Yang Y, Huang X, Liao S, Zhang F, Shi J, Duan X, Liu K. Micropulse laser trabeculoplasty on Chinese patients with glaucoma or ocular hypertension: average 35 months follow-up results. BMC Ophthalmol. 2022 Jun 4;22(1):249. doi: 10.1186/s12886-022-02477-w. PMID: 35658849; PMCID: PMC9167537.	Micropulse laser trabeculoplasty reduce IOP in patients with glaucoma or ocular hypertension for a short period, but its lowering efficiency is limited up to 6 months of the follow-up period.	
